# A Study of Reverse Causation: Examining the Associations of Perfluorooctanoic Acid Serum Levels with Two Outcomes

**DOI:** 10.1289/EHP273

**Published:** 2016-08-16

**Authors:** Radhika Dhingra, Andrea Winquist, Lyndsey A. Darrow, Mitchel Klein, Kyle Steenland

**Affiliations:** 1 Department of Environmental Health, and; 2 Department of Epidemiology, Rollins School of Public Health, Emory University, Atlanta, Georgia, USA

## Abstract

**Background:**

Impaired kidney function and earlier menopause were associated with perfluorooctanoic acid (PFOA) serum levels in previous cross-sectional studies. Reverse causation, whereby health outcomes increase serum PFOA, may underlie these associations.

**Objective:**

We compared measured (subject to reverse causation) versus modeled (unaffected by reverse causation) serum PFOA in association with these outcomes to examine the possible role of reverse causation in these associations.

**Methods:**

In cross-sectional analyses, we analyzed PFOA in relation to self-reported menopause among women (*n* = 9,192) 30–65 years old and in relation to kidney function among adults > 20 years old (*n* = 29,499) in a highly exposed Mid-Ohio Valley cohort. Estimated glomerular filtration rate (eGFR, a marker of kidney function) and serum PFOA concentration were measured in blood samples collected during 2005–2006. Retrospective year-specific serum PFOA estimates were modeled independently of measured PFOA based on residential history and plant emissions. Using measured and modeled PFOA in 2005 or 2006 (predictor variables), cross-sectional associations were assessed for eGFR and menopause (yes/no). We also analyzed measured PFOA (dependent variable) in relation to the number of years since menopause.

**Results:**

Menopause and eGFR were significantly associated with measured (trend tests: *p* = 0.013, *p* = 0.0005, respectively) but not with modeled serum PFOA (*p* = 0.50, *p* = 0.76, respectively). Measured PFOA levels increased for the first 7 years after menopause (trend test, *p* < 0.0001), providing further evidence that the observed association between measured PFOA and menopause is subject to reverse causation for this outcome.

**Conclusion:**

Our results support the conjecture that in previous studies, earlier menopause and reduced kidney function are the causes rather than the results of increased measured serum PFOA. These results suggest caution in using biomarkers in cross-sectional studies.

**Citation:**

Dhingra R, Winquist A, Darrow LA, Klein M, Steenland K. 2017. A study of reverse causation: examining the associations of perfluorooctanoic acid serum levels with two outcomes. Environ Health Perspect 125:416–421; http://dx.doi.org/10.1289/EHP273

## Introduction

Although serum biomarkers of chemical exposure are often seen as objective measures of internal dose, their use in cross-sectional studies introduces the possibility of reverse causation because measurement of both the disease and the biomarker are concurrent. Causal relationships between chemical exposure and disease may be particularly questionable if the outcome of interest can affect a chemical’s pharmacokinetics by, for example, decreasing excretion and consequently increasing accumulation in the body.

Reverse causation is a well-known concern in cross-sectional studies of outcomes and measured biomarkers including biomarkers of chemical exposure; however, demonstration of this problem is uncommon. Demonstration of reverse causation is facilitated by an alternative exposure measure, invulnerable to possible reverse causation, which can be used instead of the potentially affected exposure biomarker; often, such an alternative measure is not available. In the present work, we used measured perfluorooctanoic acid (PFOA) levels in the blood and an alternative measure, modeled PFOA levels based on estimates of external exposure. We show two specific examples of likely altered excretion rates of PFOA resulting from two health outcomes, menopause and renal function, and discuss the potential impact of reverse causation on epidemiologic analyses of each outcome in cross-sectional analyses.

PFOA, a perfluoroalkyl acid, is widely used in the manufacture of polymers, such as Teflon. PFOA is found in serum at low levels in almost all of the U.S. population (median = 4 μg/L; Calafat et al. 2007), is not metabolized (Post et al. 2012), and is slowly excreted, with a reported half-life ranging from 2.3 to 3.8 years (Bartell et al. 2010; Olsen et al. 2007).

At menopause, menstruation, which accounts for a yearly blood loss of 10–14% of blood volume (see “Calculation of yearly blood loss via menstruation” in the Supplemental Material), ceases. Without this mode of recurring excretion in chronically exposed women, rates of PFOA accumulation in blood after menopause may be greater than before menopause. Decreased serum PFOA has been shown in premenopausal versus postmenopausal women (Harada et al. 2005) and analogously in men experiencing regular blood withdrawals in the course of medical treatment (Lorber et al. 2015). Two cross-sectional studies, one of women exposed at background PFOA levels (Taylor et al. 2014) and one of women in a large, highly exposed Mid-Ohio Valley cohort (Knox et al. 2011), found positive associations between higher measured serum concentrations and earlier menopause. In exploring possible reverse causation, Taylor et al. (2014) also found a positive linear association [β = 0.07, 95% confidence interval (CI): 0.013, 0.13] between the number of years after natural menopause and the log-transformed measured serum PFOA concentration, suggesting that decreased excretion after menopause led to higher serum PFOA levels. Pharmacokinetic modeling studies at background exposure levels have noted that accounting for menstruation partially explained observed higher serum perfluoroalkyl acid (including PFOA) concentrations in men versus women (Lorber et al. 2015; Wong et al. 2014) and in girls who were not yet menstruating versus those who were (Wu et al. 2015).

Analogously, reduced renal function [calculated as estimated glomerular filtration rate (eGFR)] may result in decreased PFOA excretion and consequently, in an increased rate of PFOA accumulation in serum. Cross-sectional analyses of adults exposed at background levels (Shankar et al. 2011) and of children exposed at high levels (Watkins et al. 2013) found a positive association between lower kidney function (i.e., lower eGFR) and higher measured serum PFOA. Watkins et al. (2013) could not confirm this relationship when using an environmentally modeled exposure estimate of serum PFOA instead of a measure in blood serum.

In separate work, we longitudinally analyzed the relationships between modeled PFOA and both diagnosed chronic kidney disease (CKD, characterized by severely diminished renal function) and menopause (Dhingra et al. 2016a, 2016b). In neither case did we find evidence of association. In this context, we consider the possibility of reverse causation in the aforementioned cross-sectional associations of measured serum PFOA.

To explore reverse causation in associations of both kidney function and menopause with PFOA, we compared measured and modeled serum PFOA cross-sectionally at the time of blood draw (2005–2006) as predictors of eGFR and menopause prevalence. If PFOA exposure truly affects either outcome of interest and the association is observed with measured PFOA, then we would expect the association to also be observed when using modeled serum PFOA in place of measured serum PFOA (assuming the modeled metric is reasonably accurate). If PFOA does not alter kidney function (or, analogously, menopause prevalence), measured PFOA might still appear to be inversely associated with eGFR (or positively associated with menopause) if preexisting decreased renal function (or analogously ceased menstruation) affects PFOA excretion and hence serum levels.

We also examined yearly accumulation of serum PFOA after menopause. Owing to the loss of excretion via menstrual blood, we expected that postmenopausal women would, on average, show increasing serum PFOA for several years after menopause when compared with premenopausal women; this would also suggest that reverse causation might explain the previously observed cross-sectional associations of measured PFOA and earlier menopause.

## Methods

Our Mid-Ohio Valley community is centered around the DuPont manufacturing facilities near Parkersburg, West Virginia, where PFOA releases began in 1951 (Frisbee et al. 2009). This community was exposed to PFOA at levels higher than background exposures (median measured serum concentration was 28.2 μg/L in 2005–2006; Frisbee et al. 2009).

### Data: Surveys, PFOA and eGFR

The present cohort (*n* = 30,303) consists of Mid-Ohio Valley residents who were studied cross-sectionally in the C8 Health Project (C8HP) and longitudinally by the C8 Science Panel (C8SP). Figure S1 shows cohort formation. The present cohort consists of C8HP participants ≥ 20 years old who provided informed consent to participate in subsequent C8SP studies, which includes the present study. The Institutional Review Board (IRB) at Emory University reviewed and approved all aspects of these studies, including consent forms and surveys. The present study is covered under that IRB approval.

Our study population is the same Mid-Ohio Valley community studied cross-sectionally by Watkins et al. (2013) for eGFR, cross-sectionally by Knox et al. (2011) for menopause, and longitudinally by Dhingra et al. (2016a, 2016b) for CKD and menopause. To be included in the C8HP, conducted in 2005–2006, individuals must have been exposed for ≥ 1 year to PFOA-contaminated water from an affected water district or a contaminated private well (Winquist et al. 2013). The C8HP included a blood sample for measurement of serum PFOA concentrations (micrograms/liter) and other biomarkers, including creatinine (Frisbee et al. 2009). To calculate eGFR in adults, creatinine levels were entered into the Modification of Diet and Renal Disease study equations, a validated method for determining eGFR in Caucasian and African American adults [National Kidney Disease Education Program (NKDEP) 2014].

In C8SP surveys (2008–2011), participants provided information on demographics, health behaviors (e.g., smoking, exercise), and history of several chronic diseases. Residential history (1951–2011), including dates at each residence, was collected for each participant. Women were asked about their reproductive history, including menstrual and pregnancy history (Winquist et al. 2013). Serum PFOA concentrations were modeled for each year as part of the C8SP studies (Winquist et al. 2013).

Methods for generating year-specific (1951–2011) estimated PFOA serum concentrations have been described in detail (Shin et al. 2011a, 2011b; Winquist et al. 2013; Woskie et al. 2012) and are summarized in “Brief summary of longitudinal reconstruction of modeled serum PFOA” in the Supplemental Material. The modeled exposure estimates were generated through a set of models with inputs based on environmental information and some information collected from participants, including residential history, body weight, work history, and drinking water source and consumption rate (when provided). It is unlikely that either of the outcomes considered here would influence reporting of these factors. Concurrent (i.e., modeled in the year of blood sampling) serum PFOA estimates had a Spearman correlation with serum PFOA concentrations measured in the blood sample (2005–2006) of 0.71 (Winquist et al. 2013). All analyses used modeled or measured estimates of serum PFOA determined in 2005–2006, the time of blood draw, and included only cohort members with both measures.

### Analyses: eGFR and PFOA in Adults

Of our cohort’s 30,303 adults, 590 were excluded because they were born before 1920 (consistent with prior C8SP analyses), were missing covariates, were missing serum creatinine for eGFR determination, or were < 20 years old in 2005–2006. An additional 72 people with implausibly high eGFR (> 150) were excluded, leaving 29,641 subjects for analysis. Using linear regression, eGFR was regressed in separate models on three PFOA exposure metrics: measured serum PFOA concentrations, modeled serum PFOA concentrations, and modeled cumulative exposure (the sum of all year-specific, modeled serum concentrations up to a given year); adjustments were made for the potential confounders (see Table 1 footnote). Because Shankar et al. (2011) adjusted for high cholesterol and hypertension presumably as potential confounders, we ran the above model additionally controlling for hypertension and high cholesterol (both self-reported as physician diagnosed) as a sensitivity analysis.

**Table 1:** Results of cross-sectional regression of eGFR and PFOA (*n *= 29,641) and logistic regression of PFOA and menopause (*n *= 6,342).

PFOA exposure variable	eGFR analysis, linear regression^*a*^		Menopause analysis, logistic regression^*b*^	
	Parameter estimate ± SE	*p*-Value	OR (95% CI)	*p*-Value
Measured serum concentration (2005–2006), quintiles (reference = 1st quintile)^*c*^
2nd	–0.64 ± 0.268	0.018	1.68 (1.21, 2.35)	0.002
3rd	–1.03 ± 0.269	0.0001	1.45 (1.04, 2.02)	0.03
4th	–0.84 ± 0.271	0.0019	1.39 (1.00, 1.93)	0.05
5th	–0.98 ± 0.274	0.0003	1.58 (1.14, 2.19)	0.006
Measured serum concentration (2005–2006)^*d*^	–0.14 ± 0.07	0.03	1.09 (1.002, 1.18)	0.04
Modeled serum concentration (2005–2006), quintiles (reference = 1st quintile)^*e*^
2nd	–0.08 ± 0.268	0.77	0.98 (0.70, 1.37)	0.90
3rd	0.37 ± 0.268	0.17	1.05 (0.75, 1.45)	0.78
4th	0.21 ± 0.269	0.44	0.78 (0.56, 1.08)	0.14
5th	0.23 ± 0.271	0.41	0.92 (0.65, 1.30)	0.62
Modeled serum exposure (2005–2006)^*d*^	0.05 ± 0.058	0.43	0.98 (0.70, 1.37)	0.90
Notes: CI, confidence interval; eGFR, estimated glomerular filtration rate; OR, odds ratio; PFOA, perfluorooctanoic acid; SE, standard error. ^***a***^Potential confounders were chosen, *a priori*, from the literature and included smoking status (current/former/never), body mass index (BMI) (< 18.5, 18.5–25, 25–30, ≥ 30 kg/m^2^), education level (“less than high school (HS),” “HS diploma,” “some undergraduate education,” “bachelor’s degree or higher”), race (white vs. nonwhite), sex, and birth year [Anderson et al. 2009; Haroun et al. 2003; Centers for Disease Control and Prevention (CDC) 2007]. ^***b***^Based on a review of factors associated with menopause (Gold 2011), covariates in the model included age in 2005–2006 (linear term); parous/nulliparous status; smoking status; education; BMI in 2005–2006; and birth year (linear or 2-year categories). ^***c***^In eGFR and menopause analyses, upper cut points of 1st, 2nd, 3rd and 4th quintiles are 11.1, 19.4, 36.3, and 88.0 μg/mL, and 9.7, 17.2, 31.9, and 78.8 μg/mL, respectively. ^***d***^Serum concentrations were natural log–transformed. ^***e***^In eGFR and menopause analyses, upper cut points of 1st, 2nd, 3rd and 4th quintiles are 5.8, 11.4, 26.8, and 82.4 μg/mL, and 6.1, 11.8, 26.8, and 78.0 μg/mL, respectively.				

Each exposure variable was categorized into quintiles in the primary analysis, and the natural log–transformed continuous PFOA metric served as a trend test. To enhance clarification of the dose–response relationship between measured serum PFOA and eGFR, measured serum PFOA was further categorized into deciles. Regression coefficients and *p*-values for modeled and measured PFOA were compared to assess the potential for reverse causation.

### Analyses: Menopause and PFOA

Of 16,870 women in the present cohort, we excluded 4,455 who were < 30 years old or > 65 years old. We excluded an additional 3,223 who had incomplete menopausal history (*n* = 517), reported menopause before age 30 (*n* = 1,589), or were missing covariates. Of the remaining 9,192 women, 2,355 had experienced natural menopause, 2,850 had experienced hysterectomy, and the remainder had experienced neither.

***Cross-sectional logistic regression.*** We conducted logistic regression, analogous to prior cross-sectional analyses by Knox et al. (2011), in which reported natural menopause as of 2005–2006 was the outcome. For this analysis, we further restricted the cohort to women aged 40–60 in 2005–2006 to ensure that every age had both postmenopausal and premenopausal women, and we excluded women with hysterectomies, as done by Knox et al. (2011); 6,342 women remained after these exclusions. Exposure variables were constructed as in the eGFR analysis, using both measured and modeled exposure.

***PFOA and years since menopause.*** In the full cohort of 9,192 women, cross-sectional linear regression analyses were conducted with natural log–transformed measured serum PFOA as the outcome variable and the number of years elapsed since menopause at the time of blood draw in 2005–2006 (hereafter, referred to as “years since menopause”) as the main predictor. Women with hysterectomies were retained in this analysis, and hysterectomy was considered as equivalent to menopause in leading to a cessation of PFOA excretion via menstruation. We used either a linear variable or a 2-year categorical variable for years since menopause. The referent group (years since menopause = 0) comprised premenopausal women and those reporting that menopause occurred in the year of their blood sample collection (newly menopausal). To capture any nonlinear relationship between years since menopause and natural log–transformed measured serum levels, we used a restricted cubic spline with three knots (Harrell et al. 1988) and a linear spline with one knot. Knots were chosen to maximize fit, which was judged via *R* ^2^.

In addition to adjusting for known predictors of measured serum PFOA (Steenland et al. 2009a), we also included, as a covariate, modeled serum PFOA in 2005–2006, which added predictive value to the model because it accounted for other factors not included in the other predictors, such as detailed residential history over time. Because some known predictors are potentially related to modeled serum PFOA, we alternately removed either “known predictors” or “modeled serum PFOA” from the model as part of a sensitivity analysis. Both known predictors and modeled PFOA independently contributed to predicting measured PFOA, as judged by the model *R^2^*.

## Results

### eGFR Analyses

**Figure 1:**
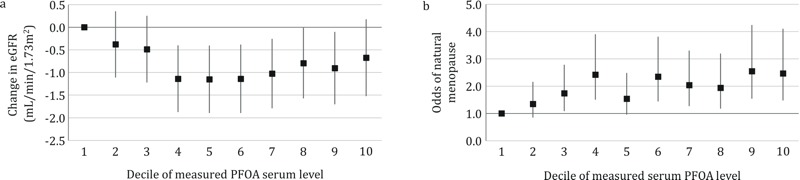
Dose–response curves (*A*) showing changes in estimated glomerular filtration rate (eGFR; milliliters/minute/1.73 m^2^) with decile of measured serum perfluorooctanoic acid (PFOA); and (*B*) showing the odds ratios for natural menopause by decile of measured serum PFOA. The first decile is the reference, and error bars indicate the 95% confidence interval.

Table S1 shows cohort characteristics for the eGFR analyses. There was a negative trend in eGFR across measured serum PFOA quintiles (Table 1; β = –0.64, –1.03, –0.84, –0.98 for 2nd–5th quintiles vs. 1st quintile, respectively). Natural log–transformed continuous measured serum PFOA (a test for trend) was negatively associated with eGFR (*p* = 0.013). Neither modeled serum PFOA nor modeled cumulative exposure showed an association with eGFR (*p* = 0.43 and *p* = 0.66, respectively). The sensitivity analysis including hypertension and high cholesterol showed very similar results to our primary analysis. Examination of eGFR and deciles of measured serum PFOA yielded a dose–response curve that decreased until the 4th decile and remained approximately flat thereafter (Figure 1a).

### Menopause Analyses

Table S2 shows cohort characteristics for the menopause analyses. We found a significant increasing trend of reported menopause with increasing measured PFOA category (trend test: *p* = 0.04; Table 1) after adjustment for age and other potential confounders, similar to the findings of Knox et al. (2011). When using modeled serum PFOA or modeled cumulative PFOA exposure instead of measured PFOA, this trend disappeared (*p* = 0.90 and *p* = 0.48, respectively). Decile categorization of measured serum PFOA as a predictor of menopause showed a dose-response curve that increased up to the 4th decile and then, with the exception of a drop at the 5th decile, remained approximately level thereafter (Figure 1b).

**Figure 2:**
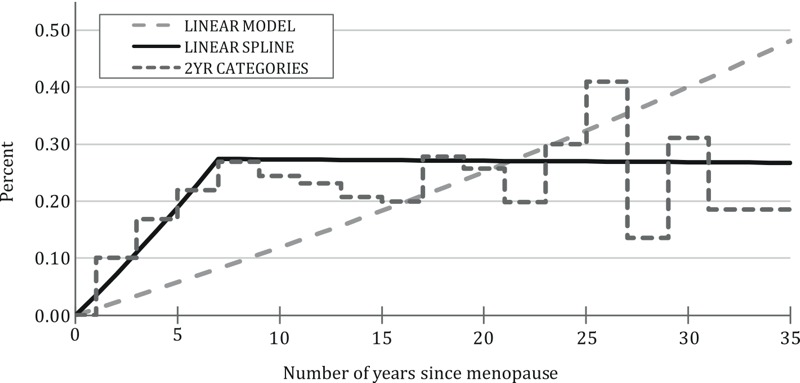
Percent increase in measured serum perfluorooctanoic acid (PFOA) as a function of years since menopause. Covariates included known predictors of serum PFOA measured in 2005–2006: smoking status; education; body mass index (BMI); growing one’s own vegetables; high cholesterol; diabetes; current residential water district; having previously lived/worked in a contaminated water district; bottled water consumption; well water consumption; birth year [2-year (2YR) categorical variable]; evidence of having worked at the DuPont plant; month of blood sample collection (categorical variables representing 2-month intervals); and modeled serum PFOA in 2005–2006.

All of our regression models of natural log–transformed measured serum PFOA in relation to years since menopause showed positive, significant associations between more years since menopause and measured serum PFOA (Figure 2). Although all exposure metrics for years since menopause had similar fit as judged by *R^2^*, varying slightly around 0.68 among metrics, the two-piece linear spline model appeared to best follow the 2-year categorization of years since menopause. The two-piece linear spline showed a steady increase of approximately 4% per year in measured PFOA with each additional year since menopause until year 7, after which no further increase was observed. Alternative sensitivity analysis models that excluded either known predictors or modeled serum PFOA produced associations that varied only slightly from our main model.

## Discussion

Our results provide evidence that reverse causation led to associations between PFOA and both renal function and earlier menopause in cross-sectional analyses. Measured serum PFOA concentrations showed a significant negative association with eGFR, but neither modeled serum PFOA nor modeled cumulative exposure showed this association. The negative association between eGFR and measured PFOA is consistent with an increase in serum PFOA that might be expected to result from decreased kidney clearance of PFOA.

Similarly, cross-sectional logistic regression models of menopause showed that measured serum PFOA was positively associated with increased risk of menopause, whereas modeled exposure metrics were not. Measured serum PFOA was on average 4% higher per year for the first 7 years after menopause than for premenopausal and newly menopausal women, after controlling for other factors known to influence serum PFOA levels in this cohort. This result is consistent with the cessation of PFOA excretion via menstruation after menopause, as proposed by Harada et al. (2007). After 7 post-menopausal years, this increased rate of accumulation appears to cease in our data, perhaps resulting from the establishment of a new steady state between intake and excretion, or perhaps caused by more error in the reported menopausal age further from the event (Rödström et al. 2005).

Causal diagrams of the modeled relationship between the available PFOA metrics and the outcomes are presented in Figure S2. These diagrams present both modeled and measured serum PFOA as imperfect measures of what might be the true etiologically relevant PFOA exposure for either menopause or eGFR if a causal association were to exist between PFOA and our outcomes. When postulated in our causal diagrams in this way, reverse causation may be thought of as measurement error of the exposure, which differs by outcome status, as discussed by Hernán and Cole (see Figure 2c in Hernán and Cole 2009).

Were a true causal relationship from PFOA to menopause to exist, neither measured nor modeled PFOA in 2005–2006 could be the etiologically relevant exposure, which often occurred years earlier, before the time of menopause. However, among menopausal women, serum PFOA modeled at the time of menopause occurrence (i.e., close in time to the potentially relevant exposure) was highly correlated with serum PFOA modeled in 2005–2006 (Spearman *R* = 0.86) and was reasonably correlated with measured serum PFOA (Spearman *R* = 0.64) in 2005–2006. If a causal relationship from PFOA to menopause existed, these observed correlations suggest that modeled PFOA would be more strongly associated with reported menopause (yes/no) in 2005–2006 than would measured PFOA. Our finding that measured exposure had a positive relationship with earlier menopause, whereas modeled exposure did not, can be considered evidence of reverse causation. An analogous argument could be made for eGFR regarding the critical time of exposure if a causal relationship existed, although unlike menopause, specific times of changes in eGFR are not known.

In these analyses, we believe that observed associations of each outcome with measured serum PFOA but not with modeled serum PFOA provide evidence that the outcome may have an impact on the measured exposure metric. However, in the presence of a true causal relationship from PFOA to either outcome, an inaccurate model of serum PFOA exposure could also yield null results for modeled exposure (e.g., nondifferential mismeasurement error), as occurred in our findings. Nevertheless, we believe that our modeled PFOA is reasonably accurate. We found a good correlation between modeled and measured PFOA in 2005–2006 (*R* = 0.71), suggesting that the model performs well. A previous study using the same modeled exposure estimates that were used in our study assessed the impact of exposure measurement error on the validity of epidemiologic models for preeclampsia (Avanasi et al. 2016). Although those authors showed that uncertainty in measurements of source data, such as observed water concentrations, might affect the serum PFOA estimates themselves, these uncertainties had little effect on the rank order of these estimates or on effect estimates for preeclampsia.

Furthermore, the modeled serum PFOA estimates used in the present analyses have allowed detection of other epidemiologic associations that are in accord with other studies. For example, these estimates have been used to reveal an association between PFOA and high cholesterol (Winquist and Steenland 2014) that had been previously shown in cross-sectional analyses using measured serum PFOA (Steenland et al. 2009b) in the same cohort; this association is now well-established in the literature (e.g., Costa et al. 2009; Frisbee et al. 2011). The modeled exposures also predicted testicular cancer in a longitudinal study in our population, an outcome that was predicted *a priori* based on animal data (Barry et al. 2013).

It should be noted that modeled estimates may also be subject to reverse causation if self-reported values of some variables in the model were influenced by health outcome status; one example is past self-reported water consumption. In this case, higher self-reported water consumption might lead to overestimates of modeled serum PFOA. However, neither of our outcomes is generally thought of by subjects as a “disease” (low GFR is generally asymptomatic, and menopause is not typically considered by the general public to be a disease). Consequently, subjects with lower kidney function or with menopause were not expected to over-report their water consumption rate as a result of the subject’s perception that high water consumption led to their “disease.”

On the other hand, our outcomes could be also associated with true water consumption. For example, there is some evidence in the literature that those with low GFR might consume less water than those with normal GFR (e.g., Clark et al. 2011; Sontrop et al. 2013). As a result, those with the disease would report lower-than-expected water consumption rates, thus biasing modeled PFOA serum levels downward among those with lower eGFR, which could in turn bias any true causal association between modeled PFOA and GFR to the null. Although we cannot exclude this possibility, the phenomenon would also result in a downward bias in measured PFOA among those with lower eGFR, which is contrary to our observation that those with lower GFR have higher measured PFOA. Water consumption is the only reasonably plausible variable used in modeling PFOA that might be affected by low eGFR, and it was but one variable among many affecting modeled PFOA. More importantly, water consumption is only a weak predictor of modeled PFOA and thus is unlikely to have any important impact. In a regression of modeled PFOA on water consumption, age, sex, and current water district, the total model *R* ^2^ is 0.41, and the partial *R* ^2^ for water consumption is only 0.03. In contrast, the partial *R* ^2^ for water district is 0.34. Residential history is a much more important variable than water consumption for determining modeled PFOA, and it is unlikely to be related to the eGFR level. Regarding menopause, we can find no data in the literature associating menopause with changed water consumption.

Nondifferential misclassification of outcomes could also result in biases to the null. For example, substantial intraindividual variation in creatinine clearance and thus in measured eGFR (Levey et al. 2015) may have biased the results of PFOA and eGFR analyses toward the null, assuming outcome mismeasurement did not differ by exposure level. This error could have affected the analyses of both measured and modeled PFOA. Self-reports of menopausal age are subject to recall bias (Rödström et al. 2005) and possible digit preference (Crawford et al. 2002). If participants were, at interview, much older than their menopausal age, measurement error may have been introduced into menopausal status (yes/no) and estimates of years since menopause, although we did not expect exposure status to affect recall of menopausal age. Such error might have particularly affected our analyses of measured PFOA and years since menopause (which included women who had been postmenopausal for many years), resulting in more error at the highest levels of years since menopause.

Despite the abovementioned limitations, our analyses suggest that elevated measured serum PFOA concentrations resulting from reduced excretion can lead to misleading cross-sectional analysis results. Nevertheless, those elevated concentrations do reflect true elevated internal dose, which may have implications for other health outcomes.

## Conclusions

Internal measures of dose may not be appropriate for some cross-sectional epidemiological studies because of reverse causation. Often, biomarkers of exposure are preferable when models yield only imprecise estimates of external environmental exposure. For outcomes that may affect the biomarker level, however, cross-sectional results should be interpreted with caution. If a reasonably accurate modeled estimate of internal exposure (dose), derived without the opportunity for distortion by individual variations in pharmacokinetics, is available, it may be preferable to use such estimates in cross-sectional epidemiological studies.

## Supplemental Material

(345 KB) PDF
