# Supplementary material for: A Study of Reverse Causation: Examining the Associations of Perfluorooctanoic Acid Serum Levels with Two Outcomes
Source: Environ Health Perspect. 2016 Aug 16;125(3):416–21. doi: 10.1289/EHP273 (PMC5332181; doi:10.1289/EHP273)
Supplement: (345 KB) PDF [file EHP273.s001.acco.pdf]

*Environ Health Perspect*
DOI: 10.1289/EHP273

**Note to readers with disabilities:** *EHP* strives to ensure that all journal content is accessible to all readers. However, some figures and Supplemental Material published in *EHP* articles may not conform to 508 standards due to the complexity of the information being presented. If you need assistance accessing journal content, please contact ehp508@niehs.nih.gov. Our staff will work with you to assess and meet your accessibility needs within 3 working days.

# Supplemental Material

# A Study of Reverse Causation: Examining the Associations of Perfluorooctanoic Acid Serum Levels with Two Outcomes

Radhika Dhingra, Andrea Winquist, Lyndsey A. Darrow, Mitchel Klein, and Kyle Steenland

## Table of Contents

Calculation of yearly blood loss via menstruation
Brief summary of longitudinal reconstruction of modeled serum PFOA
Bibliography
**Figure S1.** C8SP cohort with 2005-2006 PFOA serum measurements and estimates.
**Figure S2.** Simplified conceptual diagram of PFOA metrics and health outcomes, eGFR or menopause.
**Table S1.** Cohort characteristics of eGFR analyses (N=29,641).
**Table S2.** Cohort characteristics for menopause logistic regression (N=6,342) and 'years since menopause' (N=9,192) analyses.

## Calculation of yearly blood loss via menstruation

The expected accumulation of PFOA, when menstruation ends, can be estimated. We can assume a mean blood volume of 4.5±1.1 L (Morgan et al. 2001), approximated from observed (mean±SD) weight at age 40 (152±37 lbs.) in our cohort. Assuming a normal blood loss volume of 35–50 ml (Warrilow et al. 2004) per cycle, menstruation results in yearly blood loss (13 cycles) of ~455-650 ml/year (10-14% of blood volume), though an alternative calculation estimates greater loss of PFOA by considering the non-blood fraction of menstrual fluid (Verner and Longnecker 2015). Given that PFOA is largely bound by albumin in serum (Post et al. 2012), the later calculation includes additional loss of PFOA under the assumption (according to their commentary, substantiated in Cederholm-Williams et al. 1984) that the non-blood portion of menstrual blood contains the same concentration of albumin that is contained in blood portion of menstrual fluid.

## Brief summary of longitudinal reconstruction of modeled serum PFOA

Using mathematical modeling, historic serum PFOA concentrations were reconstructed for community participants using (1) an environmental fate and transport model to determine water source concentrations through time, (2) historical reconstruction of each individual's exposure, through air and water, based on residential history and reported water source, and (3) a single-compartment, toxicokinetic model for each individual (Shin et al. 2011a, 2011b).

For DuPont workers in the C8SP cohort (11.5%), job and department-specific occupational exposures were estimated based on ~2,000 historical serum PFOA measurements and participant work history (Woskie et al. 2012). All model estimation was done independently of PFOA values measured in 2005/06. Concurrent (i.e., modeled in the year of blood sampling) serum PFOA estimates have a Spearman correlation with serum PFOA concentrations measured in the blood sample (2005/06) of 0.71 (Winquist et al. 2013).

## Bibliography

Cederholm-Williams S, Rees M, Turnbull A. 1984. Consumption of fibrinolytic proteins in menstrual fluid from women with normal menstrual blood loss. J. Clin. Pathol. 37: 879–881.

Morgan GEJ, Mikhail MS, Larson CPJ, eds. 2001. *Morgan and Mikhail's Clinical Anesthesiology*. 3rd ed. McGraw-Hill, New York.

Post GB, Cohn PD, Cooper KR. 2012. Perfluorooctanoic acid (PFOA), an emerging drinking water contaminant: a critical review of recent literature. Environ. Res. 116:93–117; doi:10.1016/j.envres.2012.03.007.

Shin H-M, Vieira VM, Ryan PB, Detwiler R, Sanders B, Steenland K, et al. 2011a. Environmental fate and transport modeling for perfluorooctanoic acid emitted from the Washington Works Facility in West Virginia. Environ. Sci. Technol. 45:1435–42; doi:10.1021/es102769t.

Shin H-M, Vieira VM, Ryan PB, Steenland K, Bartell SM. 2011b. Retrospective exposure estimation and predicted versus observed serum perfluorooctanoic acid concentrations for participants in the C8 Health Project. Environ. Health Perspect. 119:1760–5; doi:10.1289/ehp.1103729.

Verner M-A, Longnecker MP. 2015. Comment on "Enhanced Elimination of Perfluorooctanesulfonic Acid by Menstruating Women: Evidence from Population-Based Pharmacokinetic Modeling." Environ. Sci. Technol. 49:5836–5837; doi:10.1021/acs.est.5b00187.

Warrilow G, Kirkham C, Ismail KM, Wyatt K, Dimmock P, O'Brien S. 2004. Quantification of menstrual blood loss. Obstet. Gynaecol. 6:88–92; doi:10.1016/S0015-0282(02)03638-5.

Winquist A, Lally C, Shin H-M, Steenland K. 2013. Design, methods, and population for a study of PFOA health effects among highly exposed mid-Ohio valley community residents and workers. Environ. Health Perspect. 121:893–9; doi:10.1289/ehp.1206450.

Woskie SR, Gore R, Steenland K. 2012. Retrospective exposure assessment of perfluorooctanoic acid serum concentrations at a fluoropolymer manufacturing plant. Ann. Occup. Hyg. 56:1025–37; doi:10.1093/annhyg/mes023.

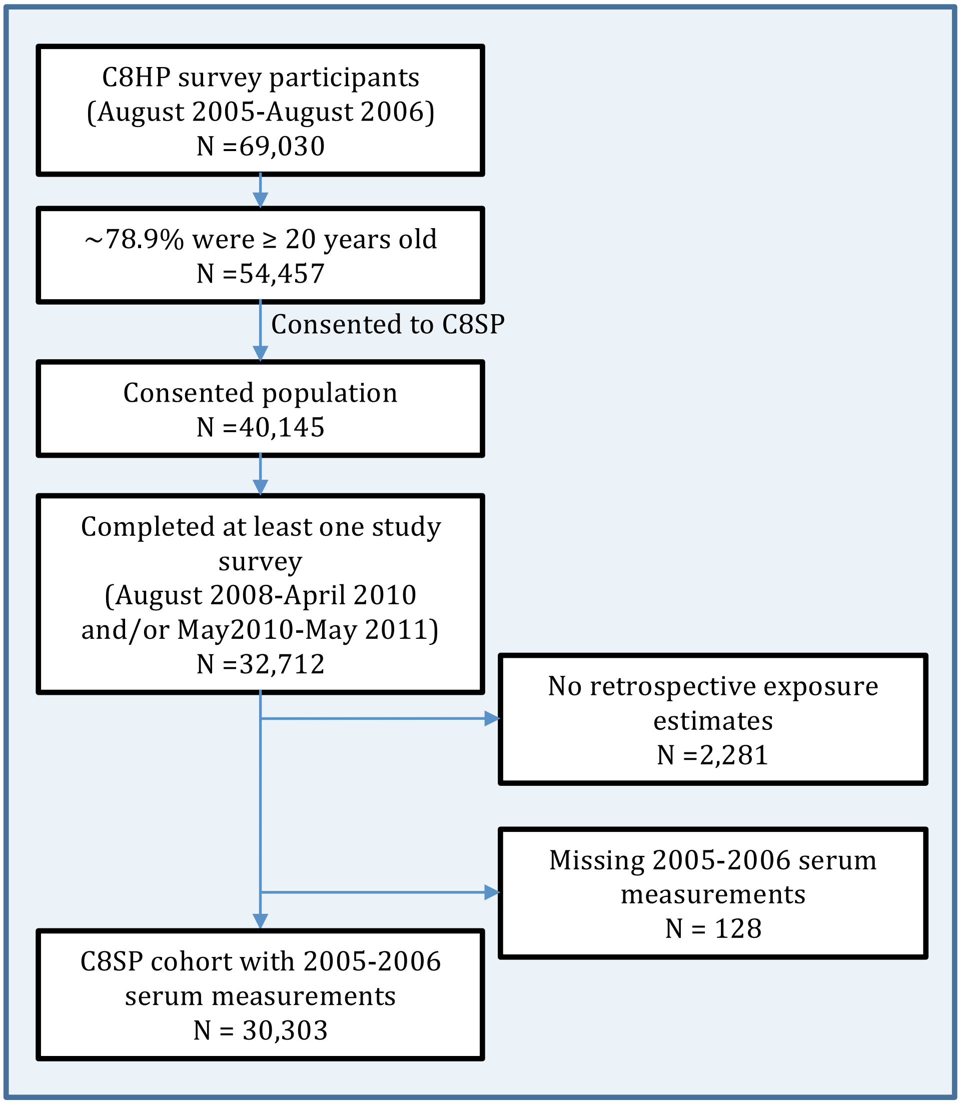


*Figure S1. C8SP cohort with 2005-2006 PFOA serum measurements and estimates.*

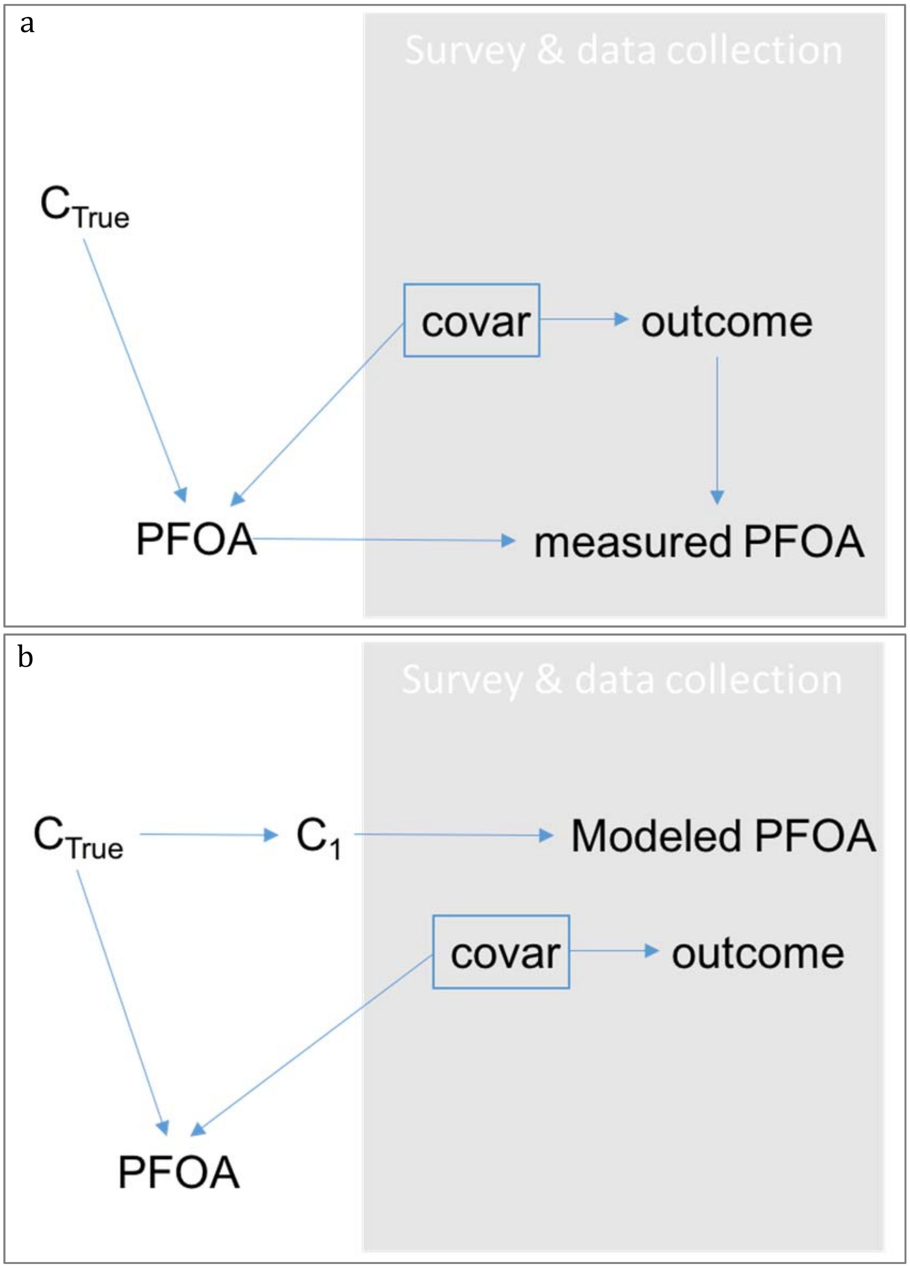


*Figure S2. Causal diagrams showing the hypothesized relationship of (a) measured and (b) modeled PFOA to either outcome. $C_{True}$ = True predictors of serum PFOA (e.g. residence, water ingestion, biological half-life); $C_1$ = modeling assumptions (e.g., reported residence, constant ingestion rate, assumed biological half-life ;PFOA = refers to the etiologically relevant exposure of PFOA occurring before data collection; modeled PFOA = modeled serum PFOA at the time of data collection; measured PFOA = Serum PFOA measured in blood at the time of data collection; Outcome = menopausal status or kidney function as measured by eGFR; covar = potential confounders of the relationship between PFOA and outcome. Nodes contained in the grey box are observed and included in either our logistic models of menopause or continuous models of eGFR.*

*Table S1. Cohort characteristics of eGFR analyses (N=29,641).*

| | | |
|---|---|---|
| **GENDER** | | |
| | **Female** | 55.6% |
| | **Male** | 44.4% |
| **HYPERTENSION** | | |
| | **No** | 66.3% |
| | **Yes** | 33.7% |
| **HIGH CHOLESTEROL** | | |
| | **No** | 76.5% |
| | **Yes** | 23.5% |
| **SMOKING** | | |
| | **Never** | 47.4% |
| | **Former** | 27.4% |
| | **Current** | 25.2% |
| **EDUCATION** | | |
| | **Less than HS education** | 8.8% |
| | **HS diploma** | 38.2% |
| | **Some undergraduate** | 34.8% |
| | **Bachelor's degree** | 18.3% |
| **BMI** | | |
| | **BMI≤18.5** | 1.4% |
| | **18.5<BMI≤25** | 26.6% |
| | **25< BMI≤30** | 34.9% |
| | **BMI>30** | 37.2% |
| **AGE AT BLOOD SAMPLING, 2005/2006** | | |
| | **Mean (SD)** | 48.2 (15.2) |
| | **Median** | 48.0 |
| **MEASURED SERUM PFOA, 2005/2006 (µg/mL)** | | |
| | **Mean (SD)** | 87.0 (281.3) |
| | **Median** | 26.1 |
| **MODELED SERUM PFOA, 2005/06 (µg /mL)** | | |
| | **Mean (SD)** | 85.2 (189.7) |
| | **Median** | 16.5 |
| **eGFR (mL/min/1.73m²)** | | |
| | **Mean (SD)** | 77.1 (16.8) |
| | **Median** | 76.3 |

*Table S2. Cohort characteristics for menopause logistic regression (N=6,342) and 'years since menopause' (N=9,192) analyses.*

| | Logistic regression analysis | Years since menopause analysis |
|---|---|---|
| **EDUCATION** | | |
| **Less than HS education** | 5.40% | 6.40% |
| **HS diploma** | 33.80% | 35.90% |
| **Some undergraduate** | 38.70% | 38.90% |
| **Bachelor's degree** | 22.10% | 18.90% |
| **PAROUS/NULLIPAROUS** | | |
| **Nulliparous** | 11.20% | 10.00% |
| **Parous** | 88.80% | 90.00% |
| **DIABETES** | | |
| **No** | 91.00% | 89.20% |
| **Yes** | 9.00% | 10.80% |
| **HIGH CHOLESTEROL** | | |
| **No** | 82.70% | 84.90% |
| **Yes** | 17.30% | 15.10% |
| **AGE CATEGORY IN 2005/2006** | | |
| **30 to 34** | 11.9 | 16.4 |
| **35 to 39** | 13.8 | 17.2 |
| **40 to 44** | 14.5 | 15.8 |
| **45 to 49** | 15.2 | 13.8 |
| **50 to 54** | 15.9 | 14.2 |
| **55 to 59** | 14.9 | 12.2 |
| **60 to 65** | 13.9 | 10.4 |
| **CURRENT WATER SOURCE** | | |
| **City of Belpre, OH** | 7.10% | 7.50% |
| **Tuppers Plains** | 13.50% | 13.60% |
| **Little Hocking Water Association** | 13.00% | 13.00% |
| **Lubeck Public Service District** | 10.80% | 11.00% |
| **Mason County** | 14.80% | 15.40% |
| **Village of Pomeroy** | 1.60% | 1.90% |
| **Well** | 4.50% | 4.30% |
| **Study area, no qualifying source** | 34.70% | 33.40% |
| **DUPONT WORKER** | | |
| **No** | 97.70% | 97.60% |
| **Yes** | 2.30% | 2.40% |
| **MODELED SERUM PFOA (μg/mL), 2005/06** | | |
| **Mean (Std. Dev.)** | 81.8 (175.0) | 84.8 (179.7) |
| **Median** | 16.3 | 16.7 |

| | Logistic regression analysis | Years since menopause analysis |
|---|---|---|
| **BMI** | | |
| **BMI<18.5** | 1.40% | 1.20% |
| **18.5≤BMI<25** | 33.10% | 31.40% |
| **25≤BMI<30** | 28.70% | 29.30% |
| **BMI≥30** | 36.80% | 39.10% |
| **SMOKING** | | |
| **Never** | 55.00% | 54.60% |
| **Current** | 24.50% | 24.30% |
| **Former** | 20.60% | 21.10% |
| **GROWS OWN VEGETABLES** | | |
| **No** | 73.10% | 73.40% |
| **Yes** | 26.90% | 26.70% |
| **DRINKS BOTTLED WATER** | | |
| **No** | 93.50% | 94.30% |
| **Yes** | 6.50% | 5.70% |
| **MONTHS OF BLOOD DRAW DURING SURVEY PERIOD** | | |
| **First 2 months** | 10.90% | 11.80% |
| **Second 2 months** | 14.10% | 14.80% |
| **Third 2 months** | 26.80% | 26.40% |
| **Fourth 2 months** | 25.70% | 25.70% |
| **Fifth 2 months** | 13.60% | 13.10% |
| **Sixth 2 months** | 8.80% | 8.20% |
| **HAVING PREVIOUSLY LIVED/WORKED IN A CONTAMINATED WATER DISTRICT** | | |
| **City of Belpre, OH** | 13.50% | 13.40% |
| **Tuppers Plains** | 19.40% | 19.40% |
| **Little Hocking Water Association** | 20.40% | 19.80% |
| **Lubeck Public Service District** | 22.10% | 22.00% |
| **Mason County** | 18.60% | 19.20% |
| **Village of Pomeroy** | 5.90% | 6.10% |
| **Tested well** | 0.10% | 0.10% |
| **MENOPAUSAL STATUS (2005/06)** | | |
| **Still menstruating** | 62.90% | 43.40% |
| **Natural menopause** | 37.10% | 25.60% |
| **Hysterectomy** | 31.00% | - |
| **MEASURED SERUM PFOA (μg/mL), 2005/06** | | |
| **Mean (Std. Dev.)** | 69.2 (195.6) | 74.6 (215.6) |
| **Median** | 21.4 | 23 |
